# Restorative effects of small urban parks: a multi-method study using eye-tracking and psychophysiological measures in Fuzhou, China

**DOI:** 10.3389/fpubh.2025.1667502

**Published:** 2025-11-24

**Authors:** Yuxi Weng, Qimei Chen, Xiabin Lin, Yuxuan Chi, Kai Li

**Affiliations:** 1 College of Design, Fujian University of Technology, Fuzhou, China; 2 College of Art and Design, Fuzhou University of International Studies and Trade, Fuzhou, China; 3 Department of Electronic Information Science, Fujian Jiangxia University, Fuzhou, China

**Keywords:** small urban parks, visual attention, attention restoration, eye-tracking technology, green space

## Abstract

**Background:**

Rapid urbanization has intensified psychological stress among urban residents. As highly accessible forms of urban green space, small urban parks play a vital role in fostering psychological resilience and restorative experiences. However, in high-density cities such as Fuzhou, the underlying restorative mechanisms of different types of small parks—along with the moderating effects of gender and specific landscape elements—remain insufficiently understood.

**Methods:**

This study adopted a multi-method approach, incorporating psychological assessment via the Perceived Restorativeness Scale (PRS), eye-tracking techniques, and physiological measurements including skin conductance level (SCL) and the low-frequency/high-frequency (LF/HF) ratio of heart rate variability. A total of 55 participants from Fuzhou were exposed to four distinct types of small urban parks—pocket parks, linear waterfront parks, community parks, and small comprehensive parks—to evaluate their restorative outcomes.

**Results:**

Both park type and gender exerted significant effects on restorative perceptions and physiological responses (*p* < 0.001). Small comprehensive parks achieved the highest PRS scores in the dimensions of being away, fascination, and compatibility, while presenting the lowest SCL and LF/HF ratios, indicative of the strongest overall restorative effects. Female participants reported higher perceived restorativeness and exhibited lower SCL values compared with male participants. The proportion of gaze fixation on vegetation was identified as the most salient positive predictor of both restorative experience and physiological relaxation. Conversely, fixation on artificial facilities and the sky showed negative predictive relationships in certain park types, while water features and traditional architectural elements displayed context-dependent positive influences.

**Conclusion:**

The restorative effects of small urban parks are jointly influenced by park type, gender, and landscape elements. Small comprehensive parks demonstrated superior performance—primarily due to their diverse and integrated natural landscapes—whereas linear waterfront parks were particularly effective in physiological regulation. Pocket parks and community parks provided comparatively weaker restorative effects, likely constrained by limited amenities and spatial configurations. Females were more sensitive to psychological perceptions (e.g., “being away,” “fascination”) and physiological responses. Natural elements (e.g., multi-layered vegetation, water bodies) served as core drivers of stable restoration, whereas the role of artificial facilities was context-dependent: traditional architecture enhanced cultural identity, while dense modern facilities potentially increased cognitive load.

## 1 Introduction

With the acceleration of urbanization, increasing population density, and a faster pace of life, urban residents are facing increasingly prominent mental health pressures. Noise, air pollution, and social pressures in high-density urban environments have been shown to significantly increase the incidence of psychological problems such as anxiety and depression (1, 2). According to the China Mental Health Survey, the lifetime prevalence of depressive disorders among adults in China is 6.8%, with major depressive disorder accounting for 3.4% (3). The Healthy China Initiative (2019–2030) proposes incorporating interventions for depression and anxiety into public health services, with the goals of raising the mental health literacy rate among residents to 30% and increasing the treatment rate for depression to 80% by 2030. Against this backdrop, urban green spaces, especially parks, are regarded as important resources for relieving stress and promoting psychological resilience. Both the Attention Restoration Theory and the Stress Reduction Theory suggest that natural environments help restore attention, alleviate mental fatigue, and enhance wellbeing (4–6). Recent studies have further shown that the accessibility and quality of urban green spaces are closely related to residents' mental health (7, 8).

As the most accessible components of urban green infrastructure in daily life, small urban parks have increasingly emerged as key settings for promoting residents' mental health, owing to their high accessibility, flexibility, and multifunctionality (9, 10). Compared with large-scale parks, small parks are more seamlessly integrated into community fabric and better cater to residents' needs for short-term, frequent interactions with nature, thereby holding irreplaceable restorative value. Nevertheless, the specific effects and underlying mechanisms through which small parks enhance psychological resilience remain to be systematically investigated empirically.

## 2 Literature review

A growing body of interdisciplinary research identifies urban parks and green spaces as important health-promoting infrastructure, contributing significantly to the psychological and physiological restoration of urban residents (11–13). Recent empirical studies and systematic reviews from diverse geographic contexts demonstrate that exposure to green spaces is significantly associated with reduced symptoms of depression, anxiety, and subjective stress, alongside improvements in wellbeing and cognitive function (14–18). Exposure duration has emerged as a key dose-dependent factor: experimental research by Hunter et al. reports that natural exposure of more than 10 min can yield statistically significant reductions in psychological stress (19), with similar findings replicated in field intervention studies in the UK and Japan (20, 21). In terms of physical health, higher vegetation coverage (measured via NDVI) has been significantly correlated with improved cardiovascular health, lower blood pressure, and reduced all-cause mortality (13, 22, 23). Moreover, green spaces play an important role in microclimate regulation by lowering surface temperatures, mitigating urban heat island effects, and improving air quality while reducing noise pollution through plant filtration and shading—thereby helping to decrease risk factors for environment-related diseases (24–26). Cross-national comparative studies further indicate that these health benefits tend to be more evident in high-density cities and low- to middle-income countries, where residents often face higher environmental stress and more limited access to green spaces (27, 28).

An important recent trend in research focuses on the health benefits of Small Public Urban Green Spaces (SPUGS). Typically located near residential areas and easily accessible on foot, these spaces can provide sustained restorative effects in the context of daily, microscale urban living (19). Peschardt and Stigsdotter's multi-site observational study in Copenhagen found that “serene” and “social” atmospheres in SPUGS significantly enhance Perceived Restorative Scale (PRS) scores, with natural features being especially important for restoring highly stressed individuals (29). Experimental studies conducted in China further suggest that high vegetation coverage, diverse plant communities, water features, and comfortable seating facilities can collectively enhance psychological fatigue recovery in pocket parks (30–32). Similarly, community and urban gardens have been reported across multiple countries to improve mental health while fostering neighborhood social capital and a sense of belonging (33–35). Zhai et al. found that micro green spaces with an area of at least 30 m × 30 m provide the most pronounced restorative effects for young adults, suggesting the presence of a spatial threshold effect (36). However, when small parks are characterized by functional monotony or lack sufficient ecological elements, they may limit both positive experiences and restorative benefits (37).

In the broader research literature, there is widespread agreement that key visual restorative factors include diverse vegetation structures, the presence of water bodies, multi-layered canopies, high sky visibility, and color richness (38–42, 107). The adoption of immersive technologies such as eye-tracking and virtual reality (VR) has enabled more precise quantification of how distinct landscape features influence visual attention patterns and emotional regulation (43–46). For example, Liu et al.'s field study demonstrated that partially open, high-naturalness urban green spaces can significantly enhance both psychological and physiological restoration by increasing visual fixation on trees and water bodies (47). Similarly, studies on window views have found that window scenes containing higher proportions of sky or green space elements can effectively alleviate psychological stress (48). However, some research suggests that natural and recreational elements are not always significant predictors of mental health (49), as their effects may be moderated by contextual factors such as perceived landscape quality, sense of social safety, and seasonality (50). Other findings indicate that natural elements (e.g., trees, grasslands, wildflowers) and historical heritage outperform artificial decorative features in highly restorative spaces, whereas extensive hard paving, visual clutter, and excessive artificialization can diminish restorative efficacy (40, 51, 52).

In summary, recent research has moved beyond the predominantly linear “green quantity–health” paradigm toward an integrated framework that incorporates landscape element optimization, exposure dose thresholds, multisensory experiences, and social equity dimensions (53). This shift provides theoretical support for understanding the multifaceted health values of small urban parks and offers a robust literature and methodological foundation for multi-method approaches, including the integration of eye-tracking with psycho-physiological measurements.

## 3 Theoretical background and hypotheses

The Attention Restoration Theory (ART), proposed by Kaplan and Kaplan (54), suggests that exposure to natural environments can alleviate directed attention fatigue by activating intrinsic cognitive resources. Directed attention is maintained through sustained voluntary effort, and prolonged use may lead to the depletion of cognitive resources, manifesting as increased distractibility, reduced impulse control, and other symptoms (4, 54). According to ART, nature triggers involuntary attention with minimal cognitive demands, allowing directed attention to rest and recover its capacity. This restorative process operates through four core dimensions: Being away, Fascination, Coherence, and Compatibility. Within the Fascination dimension, soft fascination—elicited by gentle stimuli such as vegetation and water—plays a central role by engaging passive attention under low mental load, thereby facilitating cognitive resource restoration. In contrast, hard fascination, often associated with dense artificial structures, may impose additional cognitive demands that counteract restoration (55). ART underscores the repair of cognitive resources during such restorative experiences and provides a theoretical framework for explaining how exposure to nature influences psychological outcomes, including attention and emotion.

Recent empirical evidence indicates that even small-scale urban green spaces—such as street-corner parks or pocket parks—can markedly promote attention restoration under short-term exposure (29, 56). This restorative effect has been found to be closely associated with both the landscape components and spatial configuration of these environments, encompassing physical elements (e.g., green ground cover, shrubs, trees, flower beds, water features) as well as perceptual characteristics (e.g., aesthetic preference for high-green-volume landscapes, a serene atmosphere, and a sense of safety) (57–59). Within the framework of Attention Restoration Theory (ART), such environmental factors may correspond to its core dimensions, including soft fascination (low-arousal visual stimuli that effortlessly attract attention), being away (facilitating psychological detachment from everyday stressors), and compatibility (providing support for diverse activities). Differences in park typologies may, in turn, modulate the activation patterns of involuntary attention, thereby influencing the capacity for cognitive resource restoration. Therefore, we propose the following hypotheses:

**H1: Different types of small-scale urban parks will differ significantly in their attention restoration effects, with parks containing a higher proportion of natural elements expected to have greater capacity for attention restoration**.

Stress Reduction Theory (SRT) contributes an evolutionary psychology perspective to the understanding of physiological mechanisms underlying restoration. Ulrich argued that human affinity for natural environments—such as vegetation and water bodies—derives from ancestral survival needs, for instance, vegetation signaling resource abundance and water indicating essential survival resources. This “biophilia” is thought to automatically elicit parasympathetic activation, thereby attenuating stress responses mediated by the sympathetic nervous system (6, 60). Empirical studies have shown that exposure to natural environments, through multi-channel sensory input (e.g., visual, auditory), modulates the amygdala–hypothalamus–pituitary–adrenal (HPA) axis, downregulating cortisol secretion, lowering heart rate, blood pressure, and skin conductance level (SCL), while increasing the high-frequency (HF) component of heart rate variability (HRV)—a marker of enhanced vagal tone (5, 61, 62). In contrast to Attention Restoration Theory (ART), which focuses on the cognitive mechanisms and long-term recovery of attentional resources afforded by natural environments, SRT emphasizes the physiological pathways and immediate relief characteristics of restoration, highlighting rapid stress reduction through emotional uplift and autonomic regulation under short-term exposure (63).

Taken together, these considerations suggest that different types of small-scale urban parks may vary in their capacity to activate the stress reduction pathways outlined in SRT, due to differences in the proportion of natural elements, landscape composition, and sensory attributes. For example, parks characterized by greater vegetation cover, the presence of water features, varied topography, or a quiet ambiance may more effectively enhance parasympathetic activation and autonomic balance (47, 64). Thus, the following hypothesis is proposed:

**H2: Different types of small-scale urban parks are hypothesized to differ significantly in their stress reduction effects**.

Different population groups may exhibit variations in attention restoration and stress reduction, with gender recognized as an important individual characteristic shaping restoration experiences and outcomes (65, 66). Physiologically, females have been observed to show greater increases in parasympathetic activity—as indexed by Heart Rate Variability (HRV)—along with more pronounced reductions in pulse rate and anxiety levels, following exposure to forest walking or highly vegetated urban parks (67). In contrast, males tend to demonstrate stronger cardiovascular responses and greater reductions in cortisol levels in natural environments (68, 69), accompanied by significant alleviation of work-related stress (70). Psychologically and perceptually, females generally exhibit higher sensitivity to environmental issues, stronger emotional bonds with nature (71), and a tendency to value safety, aesthetic qualities, and emotional wellbeing more highly (72, 107). For example, studies have found that static water features produce superior anxiety-reduction effects for females compared to vantage-point landscapes, whereas males derive their greatest physiological stress-relief benefits from vantage-point landscapes (73).

Gender differences manifest not only in distinct physiological recovery patterns but also in environmental perceptions and preferences. This highlights gender as a potential moderating variable in the relationship between small-scale urban parks and their restorative effects. Based on this, the following hypothesis is proposed:

**H3: Resident gender is hypothesized to exert a significant moderating effect on the capacity of small-scale urban parks to promote psychological restoration and physiological recovery**.

The Dual-Process Theory (DPT), originating from cognitive psychology, posits that humans employ two distinct yet dynamically interactive processing pathways when interpreting environmental information: System 1 and System 2 (74, 75). System 1 operates rapidly and with a high degree of automation, drawing on intuition and prior experience. It tends to elicit immediate responses to perceptually salient environmental cues—such as color, movement, or water sounds—while placing minimal demands on conscious attentional resources. In contrast, System 2 functions more slowly, relying on analytical and logical reasoning, and requires greater cognitive load to process complex or unfamiliar information (76). In the context of environmental perception and restorative studies, natural landscapes have been observed to induce low-arousal responses in System 1 through gentle sensory stimuli—for example, the “soft fascination” generated by swaying vegetation or flowing water. Such stimuli foster passive and sustained attentional engagement, while alleviating the demand on System 2 during goal-directed cognitive tasks, thereby facilitating the restoration of directed attention resources (4). However, not all environmental stimuli confer restorative benefits. Highly artificial, structurally complex, or overly salient hardscape elements—such as transportation infrastructure, densely built environments, or intense noise sources—may elicit heightened alertness or perceived threat responses via System 1, maintaining individuals in a state of high arousal. Under such conditions, System 2 must remain actively engaged in ongoing analysis and coping, thereby increasing cognitive load and mental fatigue, and potentially diminishing the restorative advantages typically associated with natural settings.

To examine the existence of such dual-system processing differences, researchers have adopted objective techniques such as eye-tracking to measure attentional patterns. Eye movement metrics (e.g., fixation duration, fixation count) provide indices of the effort involved in cognitive information processing (77, 78), thereby offering a valuable means to investigate human perceptual processes and underlying psychological activity. Berto et al. found that when viewing highly attractive natural scenes, participants exhibited significantly fewer average fixations and shorter scan path lengths compared to urban industrial scenes—indicative of the low-effort, automatic processing characteristic of System 1. Conversely, urban industrial scenes elicited more frequent fixations and broader visual exploration, reflecting the sustained engagement of System 2 and higher cognitive load (44). Similarly, Franěk et al. reported that the cognitive processing load for natural landscapes is generally lower than for urban landscapes and that eye movement patterns are significantly associated with the fractal complexity of images. More complex natural structures tended to enhance perceptual fluency and reduce visual processing effort (79). Furthermore, Valtchanov and Ellard demonstrated that natural scenes not only received higher preference ratings and longer fixation durations, but that these differences were partially attributable to low-level visual features (e.g., spatial frequency and power spectrum distribution), which were in turn linked to psychological restoration effects (80). In recent years, eye-tracking has been increasingly utilized in restorative environment research, contributing to the development of replicable experimental paradigms (81, 82). Taken together, these findings suggest that individuals' visual attention allocation between natural and artificial elements may influence the operational dominance of System 1 vs. System 2, thereby shaping both psychological and physiological restoration outcomes. Accordingly, we propose the following hypotheses:

**H4: Visual attention allocation to natural elements (e.g., vegetation, water) is hypothesized to positively predict restoration effects, whereas attention allocation to artificial elements is expected to exert context-dependent influences**.

## 4 Materials and methods

### 4.1 Study area

Fuzhou is located at the eastern end of central Fujian Province, China (25°15′-26°39′N, 118°08′-120°31′E). As one of China's “National Forest Cities,” the city has made remarkable strides in urban greening and hosts a diverse network of small urban parks. By 2025, Fuzhou's urban green space coverage ratio is projected to reach 40.33%, with a per capita park green space area of 14.93 m^2^. Characterized by a subtropical maritime monsoon climate and complex, varied topography, the region provides abundant and diverse natural conditions for the planning, layout, and plant selection of small urban parks. With a history of over 2,200 years, Fuzhou is renowned for the harmonious coexistence of its natural and cultural landscapes. Park greenspaces here are not merely critical components of the urban ecosystem but also serve as vital spaces for conserving and showcasing the city's rich historical and cultural heritage. Given these unique attributes—including its greening achievements, climatic and topographic diversity, and cultural-nature integration—Fuzhou stands as a typical and representative case for this study.

### 4.2 Park typology and stimuli

To ensure sample representativeness, 12 small urban parks within Fuzhou's urban area were selected as study sites, with considerations for urban green space distribution, population density, and functional zoning. Selection criteria included: balanced geographical distribution covering both central urban areas and surrounding densely populated regions; park areas ranging from 0.5 to 5 hectares, consistent with the definition of small parks; diverse functions (e.g., recreation, fitness, children's activities); and representativeness in construction period, maintenance status, and usage rate.

**Figure 1:**
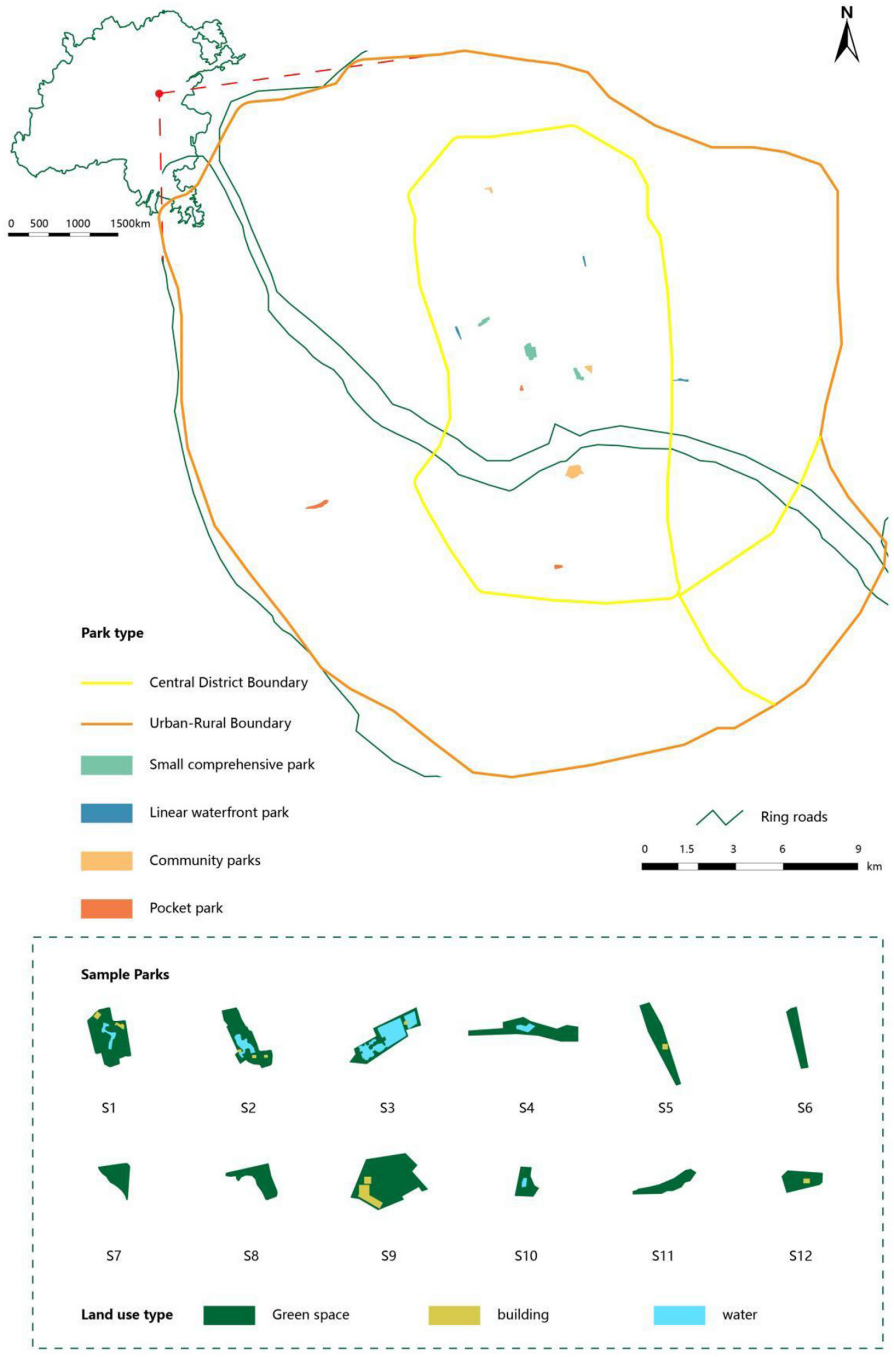
Locations of the experimental sites.

Following the Urban Green Space Classification Standards (CJJ/T 85-2017) (83), the 12 parks were classified into four types based on functional attributes, spatial structure, vegetation characteristics, and user groups: pocket parks, linear riverside parks, community parks, and small comprehensive parks. Three parks were selected per category to ensure balanced spatial distribution and coverage of diverse urban districts and functional zones. The specific distribution of sample sites is presented in Table 1 and Figure 1.

**Table 1:** Classification criteria and site information for study plots.

**Park types**	**Classification criteria**	**Site number**	**Area (hm^2^)**
Small comprehensive park	This type of park is publicly accessible and primarily functions as a recreational space, while also fulfilling ecological, landscape, cultural, educational, and emergency shelter functions. It is equipped with a range of service facilities and offers a variety of amenities, providing relatively well-developed recreational and supporting management services.	S1	4.16
		S2	3.01
		S3	4.99
Community park	This category of park is characterized by distinct thematic focus or design, complemented by purpose-built recreational and service facilities. It encompasses waterfront parks, memorial parks, sculpture parks, as well as scenic parks, urban wetland parks, and forest parks situated within urban construction zones. The green coverage ratio is at least 65%.	S4	4.82
		S5	1.89
		S6	1.21
Linear riverside park	This type of park is independently sited and equipped with basic recreational and service facilities. It primarily serves as accessible green space for daily leisure activities of residents within the surrounding community. The recommended area should be greater than 1 hm^2^.	S7	1.02
		S8	2.21
		S9	1.76
Pocket park	This type of park is independently sited, small in scale or diverse in form, and provides convenient access for nearby residents. Serving as green space with basic recreational functions, its recommended area should be greater than 0.5 hm^2^ and up to 1.2 hm^2^, with a minimum green coverage ratio of 65%.	S10	0.56
		S11	0.52
		S12	0.51

To ensure both the standardization and ecological validity of the experimental stimuli, high-quality real-scene photographs were taken at representative landscape nodes within each sampling site. The photography protocol strictly controlled environmental variables: all photographs were captured with a high-resolution digital camera during periods of stable natural lighting (09:00–11:00 AM and 14:00–16:00 PM) under sunny, rain-free conditions. For each park, 3–5 typical viewing angles (covering main functional areas) were selected, with a uniform shooting height of 1.5 meters.

**Figure 2:**
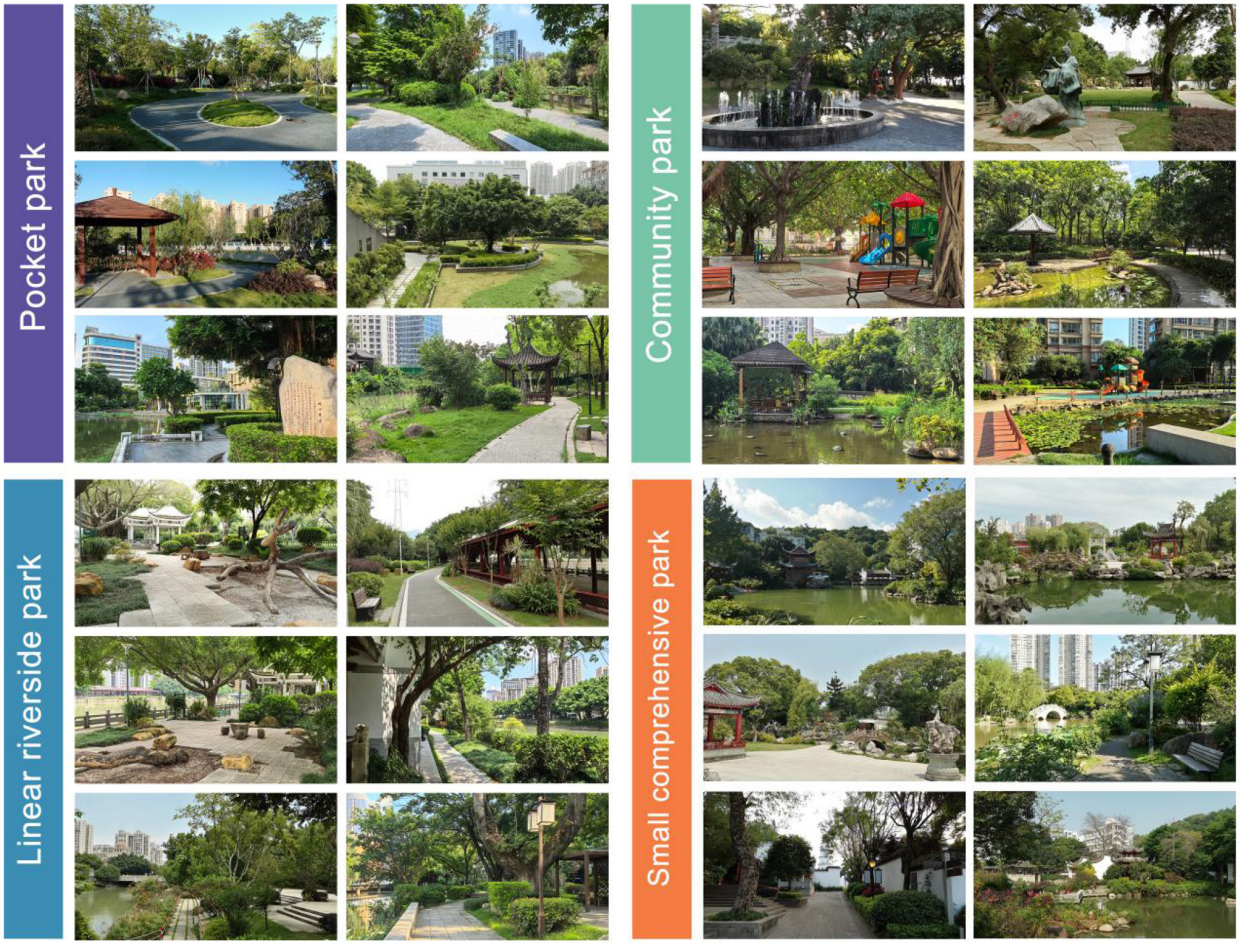
Experimental stimulus materials.

During the image processing stage, three experts in landscape architecture independently conducted a double-blind screening of representative landscape scenes (Cohen's Kappa = 0.87), indicating a high level of inter-rater reliability. For each park type, six images were ultimately retained. All selected images were standardized using Adobe^®^ software: cropped to a resolution of 3,200 × 1,800 pixels, calibrated for white balance (set to 5,500 K), and adjusted for brightness within a tolerance of ±5%. Visual distractions such as pedestrians and billboards were removed to ensure that the images emphasized the park's landscape features alone (see Figure 2 for examples of the experimental stimuli).

### 4.3 Participants

Participants were recruited through a multi-stage stratified sampling strategy combined with convenience sampling. The sampling frame encompassed the urban districts of < city>Fuzhou < /city>, China. Communities were selected via stratified random sampling based on population density. Recruitment was conducted through multiple channels, including public announcements in community bulletin boards, collaboration with local health service centers, and postings on official WeChat accounts. The target sample was stratified by age (18–30 years, 31–50 years, and ≥51 years), gender, and educational level, with proportional quotas determined according to the demographic distribution reported in the Fuzhou Statistical Yearbook (108). Inclusion criteria were defined as: permanent residents (residing for ≥1 year); aged 18–65 years; uncorrected or corrected visual acuity ≥1.0; no color blindness or color weakness; and no history of severe cardiovascular/cerebrovascular diseases, mental illnesses, or cognitive impairments. Exclusion criteria included: strenuous exercise, alcohol consumption, or use of psychoactive medications within 24 h prior to the experiment; presence of eye movement tracking interference factors (e.g., nystagmus); or inability to sit still for more than 30 min.

The required sample size was estimated using GPower^*^version 3.1.9.2. Based on effect sizes reported in comparable eye-tracking studies (*f* = 0.25) (84–86), parameters for the calculation were set as follows: significance level α = 0.05, statistical power (1 – β) = 0.95, number of groups = 1, number of repeated measures = 4, and one covariate (gender). The results indicated a minimum required sample size of 36 participants. Accounting for a 15% attrition rate observed during the pilot study, the final recruitment target was set at 55 participants. The achieved response rate was 70.5%, calculated as follows: 220 questionnaires distributed, 185 valid responses returned, with 55 participants meeting the inclusion criteria and completing the experimental procedure. The study protocol was approved by the Ethics Committee of Fujian University of Technology (approval number: FJUT-2025007). Written informed consent was obtained from all participants prior to data collection. To minimize fatigue-related confounding effects, participants completed the Pittsburgh Sleep Quality Index (PSQI) screening prior to the experiment, with only individuals scoring < 7 retained for the study.

### 4.4 Data collection

#### 4.4.1 Eye movement tracking

Eye movements were recorded using a portable eye-tracking system (sampling rate: 60 Hz; spatial resolution: 0.3° visual angle; gaze-point accuracy: 0.5°). The device captured binocular gaze data in real time while participants viewed a series of park landscape images. Prior to testing, each participant completed a 9-point grid calibration spanning the full 16:9 display area. Calibration quality was deemed acceptable if the mean error was ≤ 0.5° visual angle; recalibration was performed whenever a single attempt exceeded 1°. Participants failing calibration three consecutive times were excluded from subsequent analyses. In the present study, the calibration success rate was 92.7%. The 55 participants included in the final analysis exhibited a mean calibration error of 0.32° ± 0.11°. Real-time data validity was verified using the device's built-in quality check module. Dynamic gaze targets (randomly moving red circles) were presented to confirm tracking stability, ensuring data loss during the experiment remained below 5%. The mean data loss rate in the current study was 3.2%.

Eye-tracking analyses employed an AOI-based approach. Visual features were classified into two major categories—artificial elements and natural elements—further subdivided into seven specific AOI subclasses following established classification frameworks (46, 87, 88). The detailed AOI definitions used in this study are provided in Table 2.

**Table 2:** Division of visual interest areas.

**Visual interest area**		**Specific content**
Artificial Elements	Buildings	Bridges, Corridors, Pavilions, Towers, Walls, Residential buildings
	Man-made facilities	Protective railings, Benches, Street lamps, Trash cans, Signposts, Flower beds, Fitness equipment, Playground equipment
	Roads	Walkways, Steps
	Landscape Features	Sculptures, Landscape rocks, Rockeries, Art installations
Natural Elements	Plants	Trees, Shrubs, Glasses
	Water	Lakes, Streams, Waterfalls, Rivers, Fountains
	Sky	–

Invalid data segments attributable to blinks were identified and removed using a combined velocity–pupil diameter criterion. Specifically, potential blinks were detected when gaze velocity exceeded 30°/s, and pupil diameter exhibited an abrupt reduction of >30% relative to baseline, sustained for >100ms. Data gaps shorter than 100 ms resulting from blink removal were linearly interpolated to preserve temporal continuity. Continuous gaze data were segmented into fixations and saccades using the I–VT (Velocity–Threshold Identification) algorithm, with parameters set to a fixation velocity threshold of 30°/s and a minimal fixation duration of 100 ms. Fixations were operationally defined as gaze segments showing angular displacement < 1° and lasting ≥100 ms. To ensure data quality, outlier segments arising from excessive head movement (gaze point dispersion >2° visual angle) were manually removed. Furthermore, a 5-pixel buffer zone was applied to the boundaries of Areas of Interest (AOIs) to minimize the likelihood of misclassifying boundary fixations.

To quantify participants' visual attention distribution across distinct AOIs, the proportion of total fixation duration on each AOI relative to the total fixation duration across the entire image was calculated. The calculation formula is as follows:


$$\begin{array}{l} P_{AOI}=\frac{T_{AOI}}{T_{Total}} \end{array}$$ (1)

Here, *T*_*AOI*_ represents the total fixation duration of the participant within a specific AOI, and *T*_*Total*_ represents the total fixation duration for the entire image. This proportion reflects how visual attention is allocated to specific AOI and helps reveal how different environmental elements influence attention distribution.

#### 4.4.2 Physiological indicators

An intelligent wearable platform was employed to wirelessly acquire and analyze participants' electrodermal activity (EDA) and heart rate variability (HRV) data in real time. Key physiological indicators included the skin conductance level (SCL) and the LF/HF ratio derived from HRV.

SCL reflects the sympathetic nervous system arousal level, serving as a critical objective indicator for evaluating emotional responses and autonomic nervous activity; crucially, it remains unaffected by the parasympathetic nervous system. To account for inter-individual baseline variability, the SCL data were standardized using the formula below:


$$\begin{array}{l} X_{0}=X_{\text{emotion}}-X_{\text{calm}} \end{array}$$ (2)

Here, *X*_0_ denotes the normalized SCL data, *X*_emotion_ denotes the SCL value post-scene stimulation, and *X*_calm_ denotes the resting baseline value.

HRV was analyzed via frequency-domain methods, with the LF/HF ratio employed to reflect the regulatory balance of the autonomic nervous system—where LF denotes sympathetic nervous activity and HF denotes parasympathetic nervous activity. This ratio has been extensively utilized in evaluating psychological and physiological stress (21, 89).

#### 4.4.3 Psychological scales

The Perceived Restorativeness Scale (PRS) is based on the four core characteristics of Attention Restoration Theory (ART) proposed by Kaplan et al., and was developed by Hartig et al. (90) to assess the restorative qualities of environments and their psychological and cognitive effects (90). The Chinese version, validated by Wang et al., demonstrated good reliability (Cronbach's α = 0.80–0.91) (91). The PRS has been widely used in studies on the restorativeness of urban green spaces, effectively reflecting the influence of environmental characteristics on attention restoration (92, 93), thus supporting its selection in the present study.

### 4.5 Procedure

**Figure 3:**
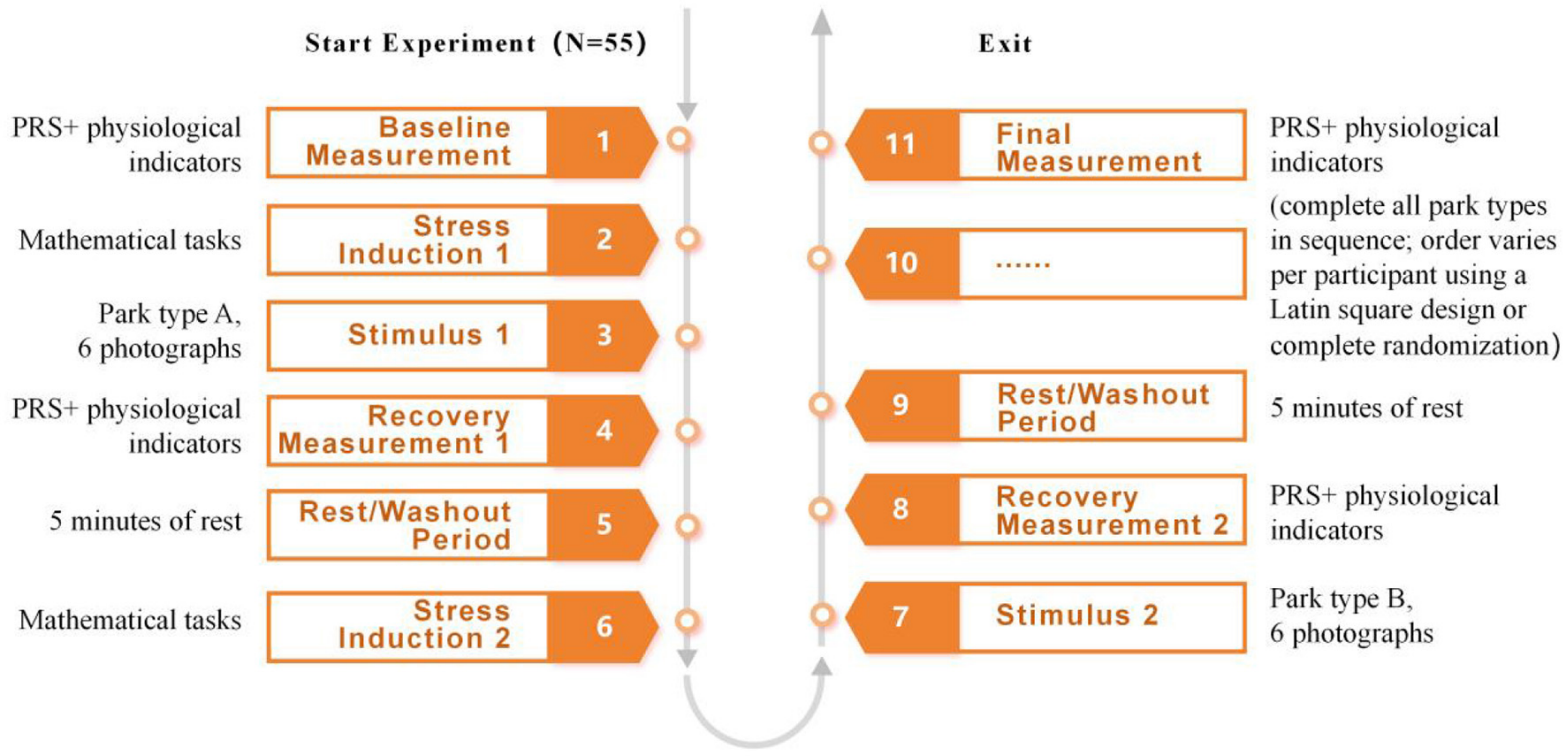
Experimental procedure.

All experiments were conducted in a quiet, well-illuminated laboratory furnished with an eye tracker, physiological signal recorders, and computer terminals. The experimental protocol is illustrated in Figure 3.

Participants initially underwent a 10-min resting adaptation phase, during which baseline measurements (encompassing subjective scales and physiological indicators) were collected. This was followed by equipment calibration and the donning of relevant instruments. After stress induction via a cognitive task (i.e., backward digit span), participants viewed images of diverse park landscape types presented in a randomized sequence. Each image was displayed for 10 s, with a 5-s inter-image interval. A 5-min washout period was implemented between distinct stimulus categories. Oculomotor and physiological data were synchronously recorded throughout the procedure. Following stimulus presentation, participants re-completed the subjective scales and subsequently dismounted the instruments. The total duration per participant was approximately 70 min.

### 4.6 Data analysis

All data were analyzed via SPSS 27.0. First, descriptive statistics were performed to compute the mean and standard deviation for each variable. Multivariate analysis of variance (MANOVA) was employed to assess the effects of distinct park types on psychological and physiological indicators. Additionally, multivariate analysis of covariance (MANCOVA) was conducted with gender included as a covariate to account for potential confounding factors. The associations between key variables were investigated via Pearson correlation analysis, and stepwise regression was subsequently applied to elucidate the effects of each predictor variable on psychological and physiological indicators. Statistical significance was defined as *p* < 0.05.

## 5 Results

### 5.1 Sample characteristics

#### 5.1.1 Demographic information

A total of 55 participants were included in this study, including 28 males (33.29 ± 9.87 years) and 27 females (38.44 ± 12.93 years). The majority of participants held a bachelor's degree. Minimal differences were observed in GHQ-12 scores and weekly frequency of nature contact across gender and educational level groups, as detailed in Table 3.

**Table 3:** Demographic information of study participants.

**Parameter**	**Category**	**N**	**Age (years)**	**GHQ - 12 Score**	**Frequency of nature contact per week**
Gender	Male	28	33.29 ± 9.87	20.29 ± 2.55	2.68 ± 1.25
	Female	27	38.44 ± 12.93	20.52 ± 4.24	3.11 ± 1.46
Education level	Primary School	3	57.33 ± 9.45	18.33 ± 5.03	4.00 ± 2.65
	Junior High School	4	51.00 ± 4.24	20.75 ± 3.96	3.00 ± 0.71
	Senior High School	3	40.67 ± 7.54	21.00 ± 0.00	2.67 ± 1.25
	Junior College	9	38.00 ± 9.42	19.33 ± 3.20	2.89 ± 0.93
	Bachelor's Degree	29	29.62 ± 8.07	19.93 ± 3.52	2.72 ± 1.33
	Master's Degree and Above	7	38.71 ± 13.96	22.71 ± 2.93	3.14 ± 1.86

#### 5.1.2 Descriptive statistics of key variables

Significant differences were observed in the proportion of fixation time on areas of interest (*P*_*AOI*_) across various park types (Table 4). Specifically, AOI-Plants consistently accounted for the highest *P*_*AOI*_ across all park types: pocket parks, linear riverside parks, and small comprehensive parks showed identical *P*_*AOI*_ values of 0.29, while community parks exhibited a slightly lower *P*_*AOI*_ of 0.21. AOI-Buildings and AOI-Artificial Facilities also contributed substantially to fixation time, with *P*_*AOI*_ ranges of 0.21–0.28 and 0.15–0.24, respectively, across all park types. In contrast, PAOI values for AOI-Sky, AOI-Roads, and AOI-Water were generally low, with most mean proportions falling within the range of 0.02–0.12.

**Table 4:** Descriptive statistics of the proportion of attention on areas of interest (*P*_*AOI*_) across various park types (*N* = 55).

**Park types**	**Area of interest**						
	**Man-made facilities**	**Sky**	**Buildings**	**Landscape features**	**Plants**	**Water**	**Roads**
Pocket park	0.16 ± 0.08	0.02 ± 0.03	0.24 ± 0.09	0.14 ± 0.07	0.29 ± 0.11	0.08 ± 0.06	0.07 ± 0.06
Linear riverside park	0.23 ± 0.08	0.03 ± 0.04	0.23 ± 0.09	0.07 ± 0.05	0.29 ± 0.09	0.12 ± 0.08	0.03 ± 0.03
Community park	0.24 ± 0.09	0.02 ± 0.03	0.21 ± 0.06	0.15 ± 0.08	0.21 ± 0.07	0.09 ± 0.07	0.08 ± 0.06
Small comprehensive park	0.15 ± 0.09	0.03 ± 0.04	0.28 ± 0.09	0.15 ± 0.06	0.29 ± 0.10	0.04 ± 0.05	0.06 ± 0.06

Significant differences were observed in the scores of attentional restoration dimensions across different park types (Table 5). Among all dimensions, “being away” consistently yielded the highest mean scores (4.36–4.77), indicating a strong perceived sense of “psychological detachment from daily routines” among participants “fascination” followed with the second-highest scores (3.83–4.45), with small comprehensive parks exhibiting the highest scores in this dimension. In contrast, “coherence” and “compatibility” dimensions showed relatively lower scores, with mean values ranging from 2.79 to 4.35. Notably, small comprehensive parks demonstrated superior performance across all dimensions, with particularly prominent scores in “being away” and “compatibility”.

**Table 5:** Descriptive statistics of scores for each dimension of the attention restoration scale (*N* = 55).

**Park types**	**Being away**	**Fascination**	**Coherence**	**Compatibility**
Pocket park	4.56 ± 1.05	4.03 ± 0.68	3.03 ± 0.62	3.81 ± 1.10
Linear riverside park	4.64 ± 0.77	3.83 ± 0.60	2.79 ± 0.59	3.59 ± 0.93
Community park	4.36 ± 0.96	4.00 ± 0.72	3.05 ± 0.90	3.95 ± 1.07
Small comprehensive park	4.77 ± 0.60	4.45 ± 0.62	3.08 ± 0.88	4.35 ± 0.89

Physiological indicators varied by park type (Table 6). Skin conductance level (SCL) was highest in pocket parks (1.36 ± 1.01) and lowest in small comprehensive parks (0.93 ± 0.70). For sympathetic-parasympathetic balance (LF/HF), linear riverside parks showed the highest mean (0.79 ± 0.37), while small comprehensive parks had the lowest (0.56 ± 0.34). Small comprehensive parks exhibited lower activation in both indicators.

**Table 6:** Descriptive statistics of physiological assessment indicators (*N* = 55).

**Indicator**	**Pocket park**	**Linear riverside park**	**Community park**	**Small comprehensive park**
SCL (μS)	1.36 ± 1.01	1.25 ± 0.80	1.04 ± 0.61	0.93 ± 0.70
LF/HF	0.67 ± 0.30	0.79 ± 0.37	0.61 ± 0.32	0.56 ± 0.34

### 5.2 Effects of park types and gender on restoration and physiological outcomes

#### 5.2.1 Main effects of park type and gender on combined restoration and physiological indicators

To investigate the effects of small park types on psychological resilience and physiological responses while controlling for gender as a covariate, multivariate analysis of covariance (MANCOVA) was performed using Wilks' Lambda as the test statistic. Results revealed significant main effects of both gender (Wilks' Lambda = 0.868, *F* = 5.302, *p* < 0.001) and park type (Wilks' Lambda = 0.741, *F* = 3.691, *p* < 0.001) on the combined dependent variables (psychological restoration dimensions and physiological measures). These findings indicate significant differences in psychological and physiological measures between genders and across small park types. Detailed statistics are presented in Table 7.

**Table 7:** Overall results of MANCOVA tests.

**Item**	**Value**	***F***	**d*f*1**	**d*f*2**	***p*-value**
Intercept	0.024	1,403.261	6	210	0.000^**^
Gender	0.868	5.302	6	210	0.000^**^
Park Types	0.741	3.691	18	594	0.000^**^

Wilks' Lambda was used for multivariate testing.

^*^p < 0.05, ^**^p < 0.01.

#### 5.2.2 Differential effects of gender and park type on restoration dimensions and physiological indicators

Univariate analysis of variance (ANOVA) was performed for each dependent variable, with results summarized in Table 8. Gender significantly affected the psychological restoration dimensions of “being away” (*F* = 10.53, *p* = 0.001), “fascination” (*F* = 8.39, *p* = 0.004), “compatibility” (*F* = 17.78, *p* < 0.001), and the physiological measure of SCL (*F* = 4.50, *p* = 0.035).

**Table 8:** Univariate analysis of variance results for main effects of gender and park type (MANCOVA).

**Dependent variable**	**Item**	**SS (Type III)**	**d*f***	**MS**	***F***	***p*-value**	**Partial η^2^**
Being away	Gender	7.672	1	7.672	10.53	0.001^**^	0.047
	Park types	4.872	3	1.624	6.69	0.010^**^	0.030
Fascination	Gender	3.582	1	3.582	8.39	0.004^**^	0.038
	Park types	11.342	3	3.781	26.57	0.000^**^	0.110
Coherence	Gender	0.460	1	0.460	0.26	0.854	0.004
	Park types	2.870	3	0.957	1.62	0.185	0.022
Compatibility	Gender	16.865	1	16.87	17.78	0.000^**^	0.076
	Park types	17.208	3	5.736	18.14	0.000^**^	0.078
SCL	Gender	2.860	1	2.860	4.50	0.035^*^	0.020
	Park types	5.993	3	1.998	9.42	0.002^**^	0.042
LF/HF	Gender	0.111	1	0.111	0.32	0.808	0.005
	Park types	1.632	3	0.544	4.78	0.003^**^	0.063

^*^p < 0.05, ^**^p < 0.01.

Park type exerted significant main effects on “being away” (*F* = 6.69, *p* = 0.010), “fascination” (*F* = 26.57, *p* < 0.001), “compatibility” (*F* = 18.14, *p* < 0.001), SCL (*F* = 9.42, *p* = 0.002), and LF/HF (*F* = 4.78, *p* = 0.003). In contrast, “coherence” showed no significant effects of gender or park type (both *p* > 0.05). These findings indicate that gender and park type significantly influence most dimensions of psychological restoration and physiological indicators.

#### 5.2.3 *Post-hoc* comparisons of park type and gender differences in restoration indicators

Subsequent Bonferroni *post hoc* tests identified significant differences across park types for the dependent variables of “fascination”, “compatibility”, SCL, and LF/HF. Specifically, the small comprehensive park exhibited significantly higher “fascination” scores than the pocket park (*p* = 0.006), linear riverside park (*p* < 0.001), and community park (*p* = 0.003). For “compatibility”, the small comprehensive park also scored higher than the pocket park (*p* = 0.031) and linear riverside park (*p* = 0.001). Additionally, the small comprehensive park showed lower SCL values compared to the pocket park (*p* = 0.045). The linear riverside park demonstrated higher LF/HF values than both the community park (*p* = 0.035) and small comprehensive park (*p* = 0.002). All other comparisons yielded no significant differences (*p* > 0.05). Detailed statistics are presented in Table 9.

**Table 9:** Bonferroni *post-hoc* pairwise comparisons of significant differences among park types.

**Dependent variable**	**Significant comparison groups (*I* vs. *J*)**	**Mean of *I***	**Mean of *J***	**Mean difference(*I* – *J*)**	***p*-value**
Fascination	1 vs. 4	4.03	4.45	−0.42	0.006^**^
	2 vs. 4	3.84	4.45	−0.62	0.000^**^
	3 vs. 4	4.00	4.45	−0.45	0.003^**^
Compatibility	1 vs. 4	3.81	4.35	−0.55	0.031^*^
	2 vs. 4	3.59	4.35	−0.77	0.001^**^
SCL	1 vs. 4	1.36	0.95	0.41	0.045^*^
LF/HF	2 vs. 3	0.79	0.61	0.18	0.035^*^
	2 vs. 4	0.79	0.56	0.23	0.002^**^

Group 1 = Pocket park; Group 2 = Linear riverside park; Group 3 = Community park; Group 4 = Small comprehensive park. Values represent means; Mean difference (I –aJ) is calculated as the mean of group I minus group J.

^*^p < 0.05, ^**^p < 0.01.

Bonferroni *post hoc* tests revealed that females exhibited significantly higher scores than males on “being away” (*p* = 0.001), “fascination” (*p* = 0.006), and “compatibility” (*p* < 0.001), and also demonstrated significantly higher SCL values (*p* = 0.038). In contrast, no significant gender differences were observed for “coherence” or LF/HF (*p* > 0.05). Detailed statistical results are presented in Table 10.

**Table 10:** Bonferroni *post-hoc* pairwise comparisons of significant differences by gender.

**Dependent variable**	**Mean (male)**	**Mean (female)**	**Difference (female – male)**	***p*-value**
Being away	4.39	4.76	0.37	0.001^**^
Fascination	3.95	4.21	0.26	0.006^**^
Coherence	3.64	4.20	0.56	0.000^**^
SCL	1.03	1.26	0.23	0.038^*^

^*^p < 0.05, ^**^p < 0.01.

### 5.3 Associations and predictive effects of visual attention allocation to landscape elements

#### 5.3.1 Correlations between proportion of visual attention allocated to landscape elements and restoration indicators

**Figure 4:**
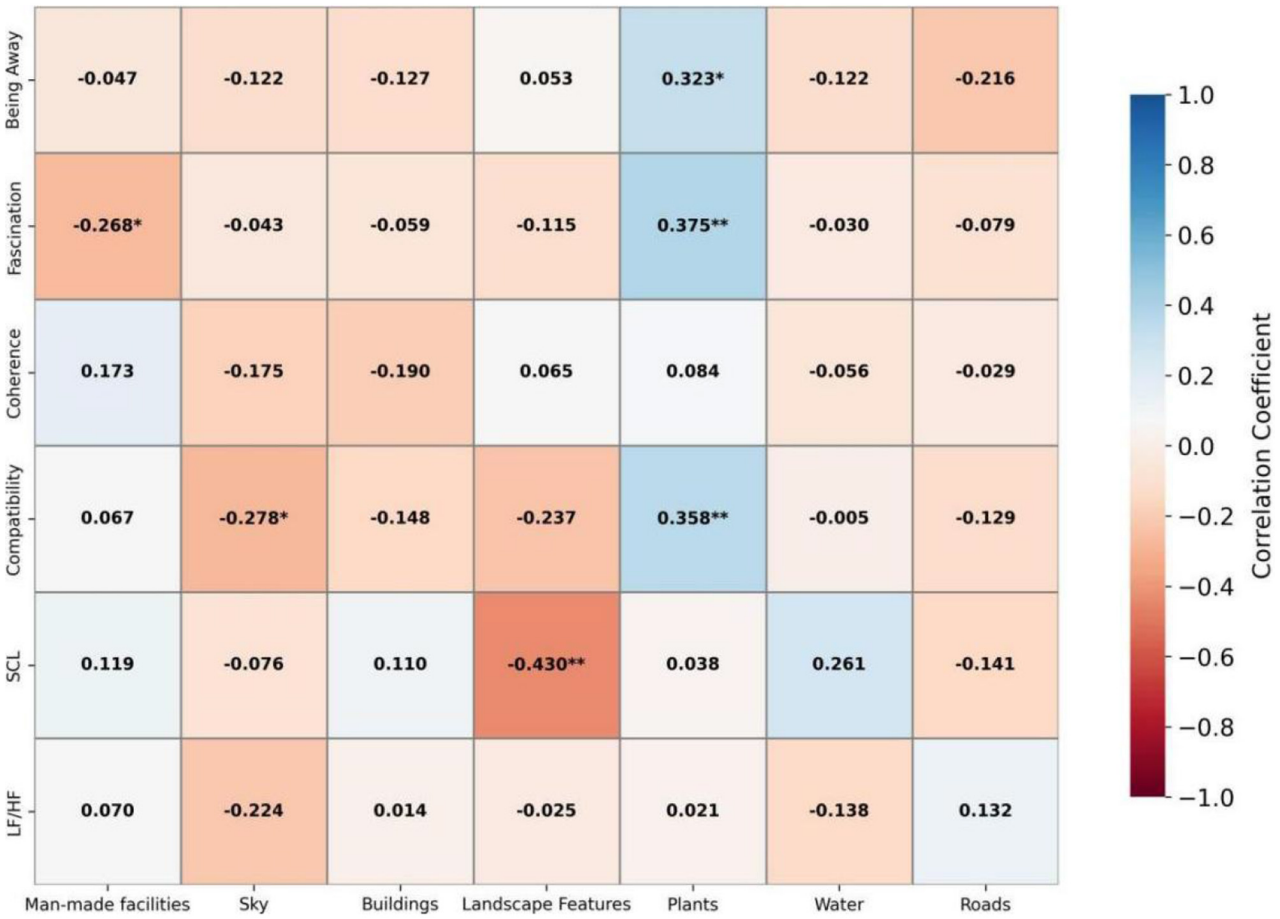
Pearson correlation analysis of small pocket park.

Pearson correlation analysis was conducted, and the results are shown in Figure 4. In pocket parks, the proportion of visual attention allocated to plants was significantly positively correlated with the ART dimensions of “being away,” “fascination,” and “compatibility” (*r* = 0.323–0.375, *p* < 0.05), indicating that attention allocation to plants helps enhance perceived restorativeness. In contrast, the proportion of visual attention allocated to artificial facilities and the sky was significantly negatively correlated with “fascination” (*r* = −0.268, *p* < 0.05) and “compatibility” (*r* = −0.278, *p* < 0.05), respectively. The proportion of visual attention allocated to landscape features was significantly negatively correlated with SCL (*r* = −0.430, *p* < 0.01), suggesting that such attention allocation may facilitate physiological recovery. Other correlations were not statistically significant. Overall, attention to natural elements appears to promote both psychological and physiological restoration, whereas attention to artificial facilities and similar man-made features may have negative effects.

**Figure 5:**
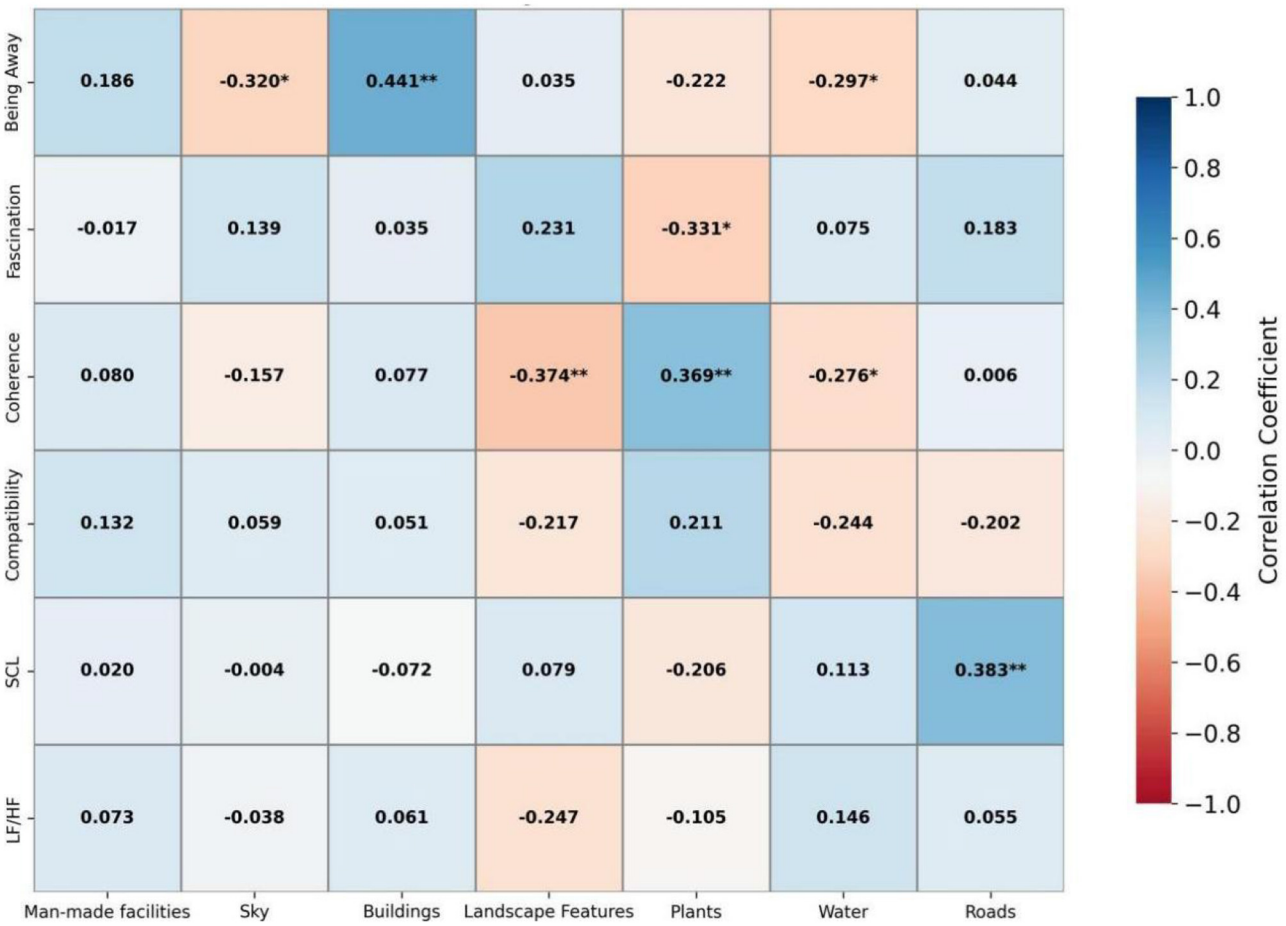
Pearson correlation analysis of linear riverside park.

Figure 5 shows the results for linear waterfront parks: the proportion of visual attention allocated to buildings was significantly positively correlated with “being away” (*r* = 0.441, *p* < 0.01), while attention to the sky and water was negatively correlated with this dimension. Attention allocated to plants was negatively correlated with “fascination” but positively correlated with “coherence.” Both landscape features and water bodies showed negative correlations with “coherence.” Notably, the proportion of visual attention allocated to roads was significantly positively associated with SCL (*r* = 0.383, *p* < 0.01). Other correlations were not statistically significant. Overall, these findings suggest that attention to buildings and plants may enhance certain aspects of restorative experience, whereas attention to elements such as roads and water bodies may be associated with negative psychological or physiological responses.

**Figure 6:**
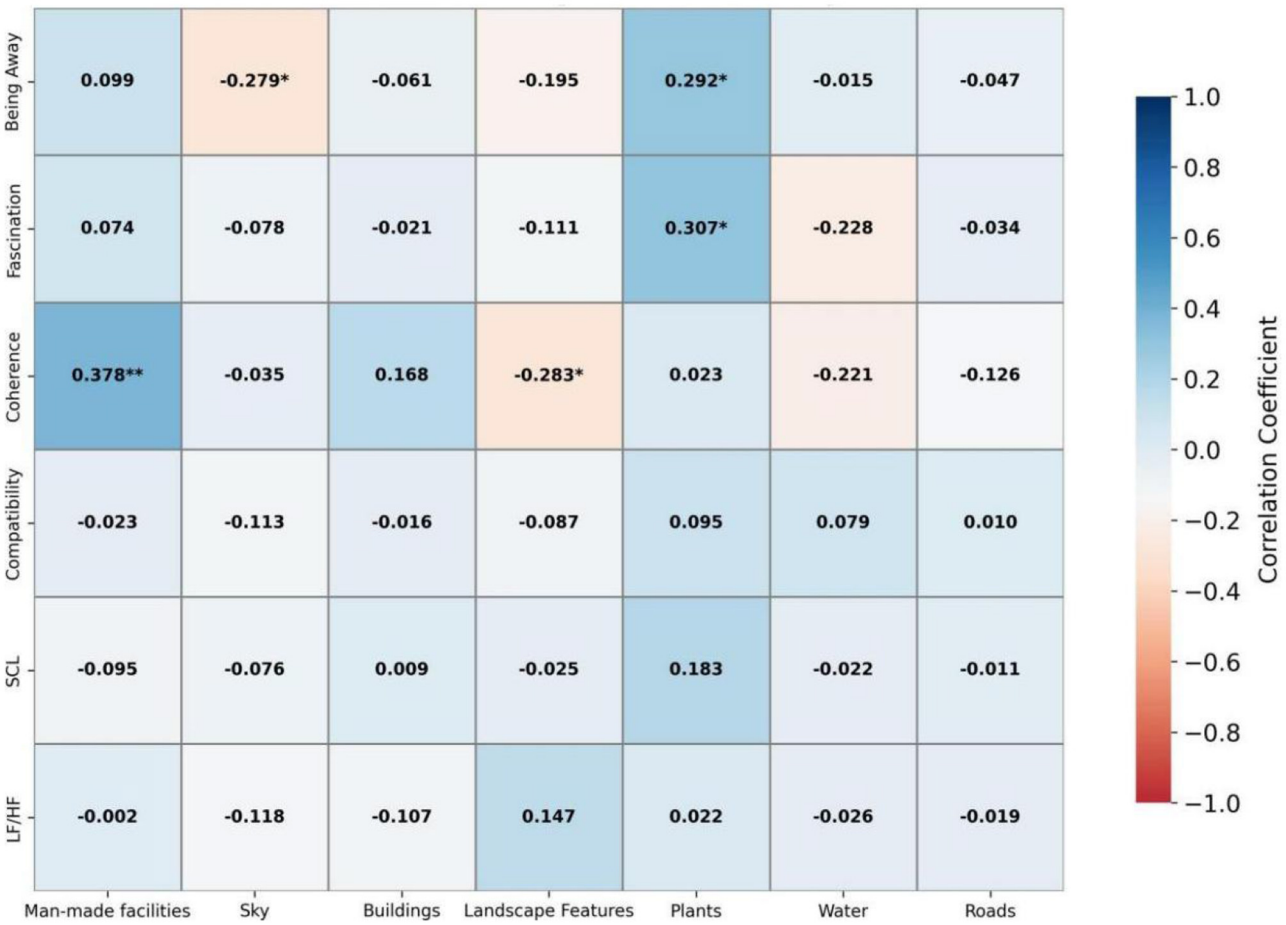
Pearson correlation analysis of community park.

Figure 6 presents the correlations for community parks: the proportion of visual attention allocated to plants was significantly positively correlated with “being away” (*r* = 0.292, *p* < 0.05) and “fascination” (*r* = 0.307, *p* < 0.05). Attention allocated to artificial facilities was positively correlated with “coherence” (*r* = 0.378, *p* < 0.01), whereas attention to landscape features was negatively correlated with this dimension (*r* = −0.283, *p* < 0.05). Attention allocated to the sky was negatively correlated with “being away” (*r* = −0.279, *p* < 0.05). No significant correlations were found with physiological indicators (*p* > 0.05). Overall, these findings suggest that attention to plants and artificial facilities may enhance restorative perceptions, while excessive attention to the sky and landscape features may have negative effects.

**Figure 7:**
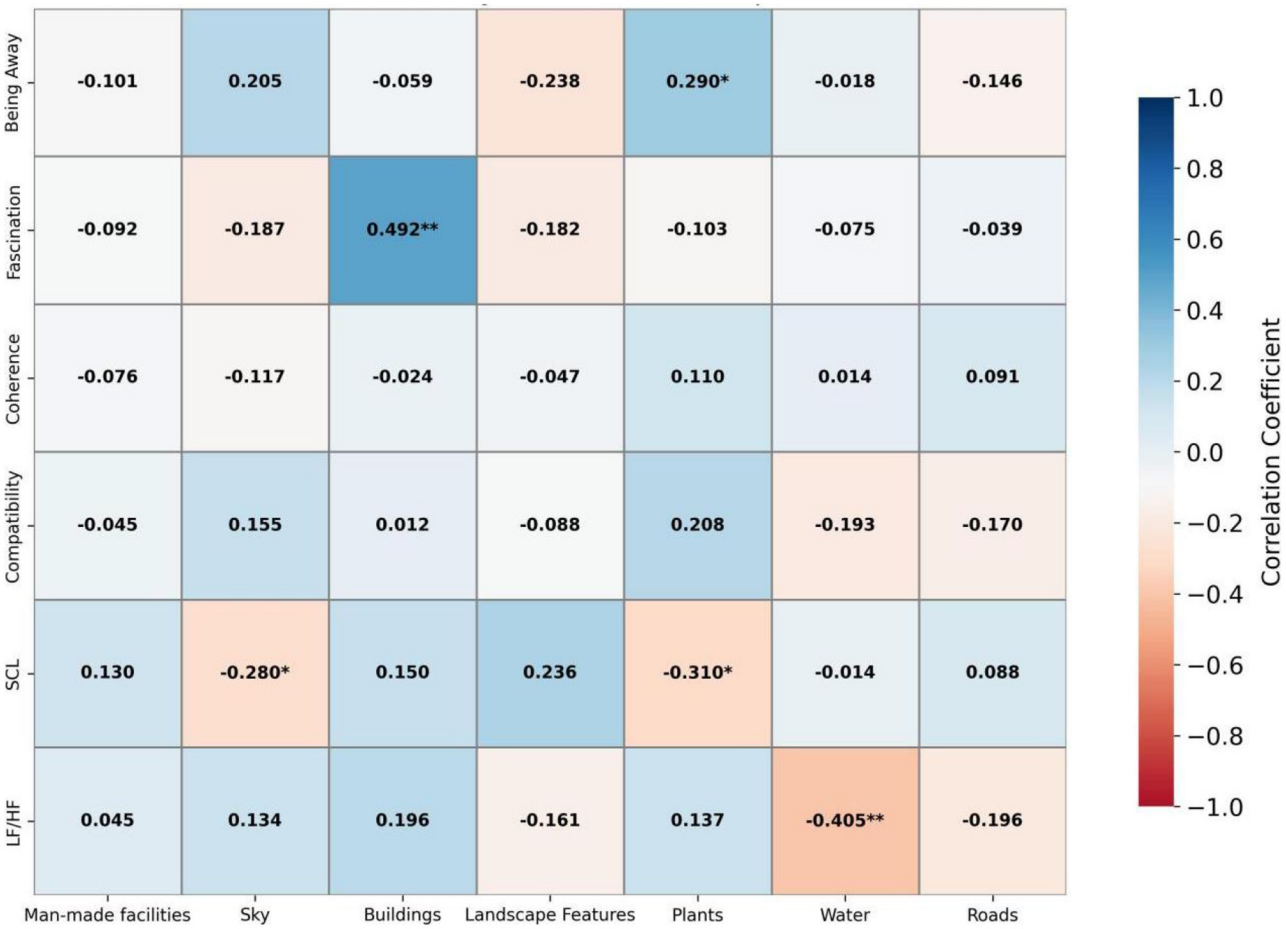
Pearson correlation analysis of small comprehensive park.

Figure 7 displays the results for small comprehensive parks: the proportion of visual attention allocated to plants was significantly positively correlated with “being away” (*r* = 0.290, *p* < 0.05), while attention to buildings was significantly positively correlated with “fascination” (*r* = 0.492, *p* < 0.01). For physiological indicators, attention allocated to plants was negatively correlated with SCL (*r* = −0.310, *p* < 0.05), attention to the sky was also negatively correlated with SCL (*r* = −0.280, *p* < 0.05), and attention to water bodies was negatively correlated with LF/HF (*r* = −0.405, *p* < 0.01). These findings suggest that focusing on plants, the sky, and water bodies may help reduce physiological arousal and promote relaxation. All other correlations were not statistically significant (*p* > 0.05). Overall, these results indicate that plant elements facilitate both psychological and physiological restoration, while buildings significantly enhance fascination.

#### 5.3.2 Predictive effects of visual attention allocation to landscape elements on restoration indicators

Stepwise regression analyses identified significant park type–specific variations in the predictive relationships between visual attention allocation to different landscape elements and both attention restoration dimensions and physiological indicators (Table 11). All regression models demonstrated acceptable statistical quality, with variance inflation factors (VIF) below 5, indicating the absence of multicollinearity. Breusch–Pagan test results yielded *p*-values greater than 0.05 in all cases, satisfying the homoscedasticity assumption and thus supporting the reliability of the estimates.

**Table 11:** Stepwise regression analysis results (*N* = 55).

**Park type**	**Dependent variable**	**Independent variable**	**unstandardized Coefficients (** ***B*** **)**		**Standardized Coefficients (β)**	***t*-value**	***p*-value**	**Adj. *R*^2^**	**Cohen's *f*^2^ (effect Size)**	**VIF**	**Breusch–Pagan (*p*)**	***F*-value**
			***B***	**Standard error**	**Beta**							
Pocket park	PRS-Being Away	Constant	3.686	0.376	–	9.807	0.000^**^	0.088	0.097 (M)	–	0.365	6.187
		AOI-Plants	2.985	1.200	0.323	2.487	0.016^*^			1.000		
	PRS-Fascination	Constant	3.374	0.238	–	14.154	0.000^**^	0.125	0.143 (M)	–	0.791	8.693
		AOI-Plants	2.238	0.759	0.375	2.948	0.005^**^			1.000		
	PRS-Compatibility	Constant	3.038	0.391	–	7.764	0.000^**^	0.168	0.202 (M)	–	0.204	6.435
		AOI-Sky	−8.596	4.036	−0.265	−2.130	0.038^*^			1.002		
		AOI-Plants	3.354	1.198	0.348	2.800	0.007^**^			1.002		
	SCL	Constant	1.803	0.867	–	2.081	0.043^*^	0.166	0.199 (M)	–	0.436	2.798
		AOI-Landscape Features	−5.619	2.119	−0.363	−2.652	0.011^*^			1.000		
Linear riverside park	PRS-Being Away	Constant	2.973	0.409	–	7.276	0.000^**^	0.253	0.339 (M)	–	0.612	10.143
		AOI-Man-made facilities	3.056	1.226	0.301	2.493	0.016^*^			1.054		
		AOI-Buildings	4.268	1.012	0.509	4.217	0.000^**^			1.054		
Community park	PRS-Being Away	Constant	3.717	0.374	–	9.934	0.000^**^	0.143	0.167 (M)	–	0.302	5.523
		AOI-Sky	−10.918	4.581	−0.301	−2.383	0.021^*^			1.005		
		AOI-Plants	4.078	1.646	0.313	2.477	0.017^*^			1.005		
	PRS-Fascination	Constant	3.370	0.284	–	11.846	0.000^**^	0.077	0.083 (M)	–	0.560	5.510
		AOI-Plants	2.980	1.270	0.307	2.347	0.023^*^			1.000		
	PRS-Coherence	Constant	2.110	0.336	–	6.276	0.000^**^	0.127	0.145 (M)	–	0.888	8.842
		AOI-Man-made facilities	3.968	1.334	0.378	2.974	0.004^**^			1.000		
mall comprehensive park	PRS-Being Away	Constant	4.241	0.253	–	16.766	0.000^**^	0.067	0.072 (M)	–	0.311	4.865
		AOI-Plants	1.805	0.818	0.290	2.206	0.032^*^			1.000		
	PRS-Fascination	Constant	3.474	0.249	–	13.975	0.000^**^	0.228	0.295 (M)	–	0.137	16.942
		AOI-Buildings	3.543	0.861	0.492	4.116	0.000^**^			1.000		
	SCL	Constant	1.620	0.298	–	5.444	0.000^**^	0.079	0.086 (M)	–	0.916	5.653
		AOI-Plants	−2.289	0.963	−0.310	−2.378	0.021^*^			1.000		
	LF/HF	Constant	0.667	0.054	–	12.289	0.000^**^	0.148	0.174 (M)	–	0.325	10.376
		AOI-Water	−2.897	0.899	−0.405	−3.221	0.002^**^			1.000		

SCL, Skin Conductance Level; LF/HF, Low Frequency/High Frequency Ratio; Effect size M, medium (Cohen's f^2^: 0.02–0.35); VIF, Variance Inflation Factor (values < 5 indicate no substantial multicollinearity).

^*^ and ^**^denote statistical significance at the 0.05 and 0.01 levels (two-tailed), respectively.

In pocket parks, greater visual attention to plant landscapes (AOI-plants) was a positive predictor of being away (adjusted *R*^2^ = 0.088, Cohen's *f*^2^ = 0.097) and fascination (adjusted *R*^2^ = 0.125, *f*^2^ = 0.143). In combination with attention to sky elements (AOI-sky), plant landscapes also positively predicted compatibility (adjusted *R*^2^ = 0.168, *f*^2^ = 0.202). In contrast, higher attention allocation to landscape features (AOI-landscape features) negatively predicted skin conductance level (SCL) (adjusted *R*^2^ = 0.166, *f*^2^ = 0.199).

For linear riverside parks, attention to architectural landscapes (AOI-buildings) and artificial facilities (AOI-artificial facilities) both positively predicted being away (adjusted *R*^2^ = 0.253, *f*^2^ = 0.339).

In community parks, visual attention to plant landscapes positively predicted being away, whereas attention to sky elements (AOI-sky) negatively predicted the same dimension (overall adjusted *R*^2^ = 0.143, *f*^2^ = 0.167). Plant landscapes also positively predicted fascination (adjusted *R*^2^ = 0.077, *f*^2^ = 0.083), while artificial facilities positively predicted coherence (adjusted *R*^2^ = 0.127, *f*^2^ = 0.145).

Results for small comprehensive parks indicated that attention to plant landscapes positively predicted being away (adjusted *R*^2^ = 0.067, *f*^2^ = 0.072), and architectural landscapes positively predicted fascination (adjusted *R*^2^ = 0.228, *f*^2^ = 0.295). Plant landscapes negatively predicted SCL (adjusted *R*^2^ = 0.079, *f*^2^ = 0.086), while water landscapes (AOI-water) negatively predicted LF/HF heart rate variability ratio (adjusted *R*^2^ = 0.148, *f*^2^ = 0.174).

In summary, attention allocation to plant landscapes consistently exhibited a stable positive effect (with moderate effect sizes) on attention restoration dimensions across all park types. In contrast, the effects of elements such as architecture, artificial facilities, sky, landscape features, and water showed context-dependent variations, differing by park type and specific indicators.

## 6 Discussion

### 6.1 Differential effects of small urban park types on psychological and physiological restoration

This study found that park type significantly affects psychological restorativeness—including being away, fascination, and compatibility—as well as physiological indicators (skin conductance level and the ratio of low-to-high frequency heart rate variability), thereby validating Hypothesis 1 and Hypothesis 2. These findings are consistent with previous research indicating that different types of natural environments can influence restorative outcomes through combinations of spatial forms and natural elements (64, 92). Furthermore, this study adds empirical evidence from high-density cities in southern China and elucidates restorative mechanisms in spatially constrained settings within the local context.

Psychologically, small-scale comprehensive parks scored higher in fascination and compatibility, while physiologically, they exhibited the lowest skin conductance level (SCL) and a reduced LF/HF ratio, indicating a pronounced relaxation effect. Despite a similar plant-gazing proportion to pocket parks (29%), their composite “plant–water–traditional architecture” configuration has the potential to fulfill multiple restorative attributes within the Attention Restoration Theory (ART) framework, including higher extent, soft fascination, and greater compatibility. Dynamic water features and multi-layered evergreen canopies may induce low–cognitive-load automatic attention processes (“soft fascination”), whereas winding pathways, corridors, and partially explorable spaces may sustain moderate engagement of System 2 (analytical processing) without triggering excessive cognitive fatigue. This balanced interplay between passive attraction and active exploration likely underpins the superior restorative performance of comprehensive parks compared to single-element parks. Nonetheless, this mechanistic inference, while consistent with dual-process theory, warrants further validation through experimental or process-tracing approaches in future research.

In contrast, pocket parks, with a mean area of only 0.53 hectares and a limited diversity of landscape elements, offer less immersive experiences in “being away” and “fascination” compared to comprehensive parks. They also exhibit significantly higher skin conductance levels (SCL), indicating weaker physiological relaxation effects. Although previous studies have demonstrated that limited natural elements in pocket parks can benefit both psychological and physiological health (30, 36, 58), our findings suggest that when the natural-to-built ratio is low and artificial features are overrepresented, these benefits may be attenuated and, in some cases, offset by increased cognitive load, thereby reducing perceived restorativeness. For severely space-constrained pocket parks, restorative potential may be enhanced through “high-density, high-efficiency” micro-design strategies, such as increasing vertical greening, incorporating small-scale water features, and optimizing the distribution of artificial facilities to achieve a more balanced integration of natural and built elements.

Notably, linear waterfront parks achieved the most favorable LF/HF ratio, indicating better autonomic nervous system (ANS) balance. This outcome is consistent with Stress Reduction Theory (SRT), which posits that proximity to water environments facilitates relaxation through multisensory stimulation (94, 95). We further hypothesize that these benefits may also derive from the environmental predictability and spatial legibility afforded by continuous pedestrian pathways and directional landscape layouts in Fuzhou's waterfront spaces, as well as from regional cultural symbols (e.g., traditional wharf imagery) that reinforce place attachment and enhance emotional stability. Nevertheless, these interpretations should be interpreted cautiously, and future studies could incorporate qualitative interviews and longitudinal tracking to further examine these mechanisms.

Furthermore, our results indicate that psychological and physiological indicators of restoration do not necessarily change in parallel across different park types. For example, the physiological advantages observed in linear waterfront parks—such as improved autonomic balance—may not correspond to overall superiority in psychological outcomes. This discrepancy may reflect temporal dynamics between psychological perception and physiological regulation, underscoring the importance of multi-dimensional and simultaneous measurements rather than relying on a single indicator to infer restoration effects.

Overall, small parks in high-density urban contexts demonstrate marked heterogeneity in restorative outcomes, underscoring the need for type-specific design and planning strategies to promote both psychological and physiological wellbeing. For comprehensive parks, it is advisable to preserve their multi-element, composite landscape configurations; for pocket parks, design efforts should focus on maximizing natural elements and optimizing the spatial arrangement of built facilities within limited areas; and for linear waterfront parks, the intrinsic benefits of water features should be retained while incorporating additional interventions to strengthen psychological engagement. These strategies could inform context-sensitive public health interventions through urban green space design.

### 6.2 Moderating role of gender in park-based restoration

This study supports Hypothesis 3 by showing that females scored significantly higher than males in certain psychological restoration dimensions, specifically sense of being away, fascination, and compatibility, as well as in skin conductance level (SCL). These results corroborate previous findings that females are more likely than males to experience positive emotional responses in natural environments (66, 96).

Notably, in the present study, females exhibited higher skin conductance levels (SCL)—a physiological indicator generally associated with heightened arousal—while simultaneously reporting greater subjective restoration. Such a pattern is relatively uncommon in environmental psychology, where restorative effects are often linked to reduced physiological arousal (97). Nevertheless, previous studies suggest that in contexts dominated by positive emotional states, moderate arousal may signal increased environmental engagement and vitality (98, 99) rather than a stress response. According to positive emotion theory, mild to moderate sympathetic activation can expand attentional scope and cognitive resources, thereby amplifying subjective pleasure (100). Because this study did not directly assess the valence of emotional arousal, the interpretation of this pattern remains speculative; however, it may indicate a tendency toward concurrent activation of emotional and attentional systems in females during natural exposure. Future research could incorporate eye-tracking metrics (e.g., fixation duration on vegetation) to quantify gender-specific visual processing of natural elements, building on methods used in cognitive studies (101).

These findings highlight the importance of gender-sensitive approaches in urban park landscape design. Beyond facilitating relaxation, parks should incorporate a range of features that can elicit positive arousal, particularly for populations more sensitive to heightened activation, such as females, thereby maximizing their psychological restoration potential under conditions of positive affect.

### 6.3 Influence of natural and artificial elements on restoration outcomes

This study supports Hypothesis 4, indicating that a higher proportion of visual attention directed toward natural elements (e.g., vegetation, water) generally correlates with greater psychological restoration and more favorable physiological responses, whereas the influence of artificial elements appears to be more context-dependent. Beyond confirming the restorative advantages of natural elements, these findings highlight the limitations of a simplistic “natural vs. artificial” dichotomy in explaining restoration effects within small parks in high-density urban settings. Instead, they underscore the need to examine how specific elements interact within particular spatial and experiential contexts.

Plants consistently serve as the most stable restorative element across all park types, with higher proportions of visual attention to vegetation significantly associated with elevated “being away” and “fascination” scores, accompanied by concomitant decreases in skin conductance level (SCL), indicative of reduced physiological arousal. These findings accord with the “soft fascination” construct in Attention Restoration Theory (ART), which proposes that natural features with low cognitive demands can passively capture attention and facilitate directed-attention recovery (4, 63). Previous research similarly suggests that greater plant diversity and enhanced spatial layering improve residents' perceived restoration and extend their length of stay (22, 33, 38). Empirical evidence from high-density southern Chinese cities in this study reconfirms these effects and underscores the critical design imperative of preserving a strong green core in small-scale parks.

The restorative effects of sky and water elements demonstrated marked type-specific and context-dependent patterns. In community parks, sky-directed visual attention was negatively associated with “being away,” diverging from earlier findings suggesting that expansive sky views facilitate psychological relaxation (40). This discrepancy may be explained by frequent obstruction of the sky background by surrounding high-density buildings in our study sites, which fragmented the visual field and limited its capacity to provide a continuous sense of visual escape. In contrast, in small-scale comprehensive parks, water-directed visual attention exhibited a significant negative association with LF/HF, aligning with established evidence from blue space research on the physiological benefits of aquatic environments (95).

Although certain artificial elements may hinder restoration, not all artificial landscape features exert negative effects on emotional states and restorative outcomes (102). Instead, their influence appears to depend on specific spatial functions and the degree of cultural integration. Firstly, in linear waterfront parks, the fixation ratio on architectural and artificial facilities was found to positively predict “being away.” This may reflect the role of moderately scaled, contextually integrated structures in enhancing spatial legibility, sense of orientation, and perceived safety (29, 103, 104). Similarly, in community parks, artificial facilities primarily contributed to “coherence.” Sports and leisure structures in these contexts may strengthen perceptions of environmental structure and increase opportunities for social interaction, in line with previous research findings (36). However, in certain contexts—such as pocket parks with densely concentrated facilities—the fixation ratio on artificial elements was negatively correlated with restorative experience and even associated with elevated skin conductance levels (SCL). This pattern suggests that excessive artificial structures or heterogeneous visual elements may increase visual complexity and sensory demands, thereby diminishing psychological restoration effects. Such findings align with earlier studies demonstrating that the tranquility and preference for natural environments decrease markedly with rising levels of visual clutter, defined as the density and heterogeneity of visual stimuli within a scene (105).

An especially salient finding relates to the cultural dimension of architectural elements. Landscapes featuring traditional garden architecture—such as antique-style verandas and pavilions—were associated with significantly higher scores on psychological restoration dimensions. This effect appears to derive not only from their aesthetic appeal but also from the evocation of place identity (106), thereby amplifying restorative experiences through emotional and cultural resonance. Such results introduce a valuable cultural lens for future restorative-environment research: in regions imbued with rich historical and local cultural symbolism, architectural elements may function as active restorative catalysts through contextual resonance, rather than merely as potential sources of visual distraction.

In conclusion, this study refines and extends the prevailing view that natural elements generally outperform artificial ones in promoting restoration, demonstrating that their restorative effects are both type- and context-dependent. While prioritizing natural features as the dominant component, artificial elements should be strategically incorporated to enhance spatial accessibility, legibility, and cultural belonging. Such an approach may achieve a balance among diverse restorative needs within limited spaces, thereby offering practical guidance for the design of high-density urban environments.

### 6.4 Design recommendations

The following evidence-based recommendations are proposed for the design and renovation of small urban parks in Fuzhou and analogous cities:

**Enhancing the diversity of natural elements and attending to environmental perceptual details**. It is recommended that small-scale parks adopt multi-layered arrangements of trees, shrubs, and groundcover to provide variation in seasonal appearance, color, and texture, thereby enhancing sensory engagement. In practice, planting species with differing flowering periods or foliage colors along pathways can extend visual interest over time, while purposefully designed open vistas can enhance landscape attractiveness and encourage spatial exploration.

**Aligning artificial facilities with park types and functional purposes**. In community parks, moderate increases in seating, fitness equipment, and other amenities can serve frequent users while preserving spatial continuity. In pocket parks, limiting the extent of artificial elements and ensuring that facility materials and color schemes harmonize with vegetation and topography can help avoid an overly artificial appearance.

**Integrating local cultural elements to strengthen emotional connections**. Built structures and landscape features should align with the overall environmental style by incorporating traditional local motifs, materials, or symbols, thereby enhancing distinctiveness and fostering a sense of belonging. Thoughtful application of traditional aesthetics can further strengthen residents' emotional resonance and place attachment.

**Addressing gender-specific needs**. The design process should explicitly account for gender-based differences in preferences. In particular, for female users, enhancing perceived landscape safety (e.g., maintaining visual openness, providing adequate lighting) and incorporating refined aesthetic features can strengthen positive restorative experiences.

### 6.5 Limitations and future research directions

Several limitations of this study should be acknowledged. Firstly, the cross-sectional design hinders the establishment of causal relationships between park characteristics and restoration effects. The current results merely reflect associations at a specific time point and cannot reveal dynamic change patterns or dose-response relationships under long-term exposure. Secondly, the laboratory environment exhibits significant ecological validity constraints: static image stimuli lack the multisensory interactions (e.g., soundscapes, olfactory cues) and dynamic behavioral experiences (e.g., walking, social engagement) inherent in real park settings, potentially underestimating the moderating effects of complex environments. Finally, there are temporal precision discrepancies in the simultaneous collection of psychological scales and physiological indicators. Additionally, the sample is predominantly composed of middle-aged and young urban residents, with geographical restriction to Fuzhou's urban area, which may limit the generalizability of the findings.

Future research could address these constraints in several ways. Firstly, adopting longitudinal and experimental designs—such as seasonal tracking or randomized controlled trials with varied park-based interventions—could help establish causal relationships and quantify dose–response functions between specific landscape elements and restoration outcomes. Secondly, enhancing ecological validity through technological integration, including mobile eye-tracking and wearable physiological monitoring in real park environments, could enable synchronized capture of visual attention patterns and stress responses, while also documenting the influence of dynamic behaviors (e.g., lingering viewing, route choice) on restoration processes. Thirdly, expanding sample diversity to include older adults, children, and high-stress occupational groups, coupled with cross-scale analytical approaches such as spatial econometric modeling, could link micro-level individual outcomes with macro-level park distribution characteristics. Furthermore, exploring contextual moderators—including extreme weather conditions and cultural background differences—would strengthen the transferability and practical applicability of the findings across diverse environmental and social contexts.

## 7 Conclusions

This study investigated the promotive effects of different types of small urban parks in Fuzhou on residents' psychological restoration and physiological relaxation. The findings revealed that park type, gender, and specific landscape elements significantly influence restoration outcomes. Small comprehensive parks exhibited optimal performance in both psychological restoration (e.g., “fascination” and “compatibility”) and physiological relaxation (e.g., reduced skin conductance levels and stabilized heart rate variability), attributed to their integrated composite landscapes of plants, water bodies, and traditional architecture. This structural combination simultaneously activates the “soft fascination” pathway of Attention Restoration Theory (ART) and the parasympathetic regulatory pathway of Stress Reduction Theory (SRT). Linear riverside parks showed distinct advantages in autonomic nervous regulation, particularly in balancing heart rate variability. Pocket parks, despite comparable plant-gazing durations to comprehensive parks, were limited by their small size; excessive artificial facilities potentially weakened restoration efficacy. Community parks featured prominent social functions, but dense artificial amenities increased cognitive load, resulting in weaker “coherence” experiences. Females displayed greater restoration benefits in psychological dimensions (e.g., “being away” and “fascination”) and skin conductance responses, suggesting that gender disparities may influence restoration experiences through differences in emotional perception sensitivity and autonomic nervous regulation. Plants (e.g., multi-layered vegetation configurations) consistently enhanced psychological restoration and physiological relaxation across all park types. The role of artificial facilities was context-dependent: Traditional architecture (e.g., archaic-style pavilions) enhanced “fascination” through cultural identity. Dense modern amenities (e.g., fitness equipment) potentially increased cognitive load, particularly in pocket parks, where they exacerbated reductions in restoration efficacy.

Several design strategies are proposed to optimize the restorative potential of small urban parks in high-density cities: Implement multi-layered vegetation configurations (arbor-shrub-herb layers) integrated with seasonal variations and water landscapes to strengthen “soft fascination” and prolong the visual interest cycle. In community parks, incorporate resting and fitness amenities in moderation, ensuring that the proportion of artificial facilities does not exceed 20% of the visible area. Pocket parks should prioritize high vegetation coverage, ensuring that facility materials and color schemes blend seamlessly with the surrounding natural environment. Incorporate traditional cultural symbols (e.g., Fuzhou's “saddle-shaped” roof forms) and landscape ornaments to reinforce cultural identity and “place attachment”. To address female preferences, optimize landscape permeability (e.g., open lawns, low hedges) and detailed aesthetic appeal (e.g., flower combinations). These recommendations aim to realize the green space value of “small space, big health” in high-density cities through the principles of “maximizing natural elements, minimizing artificial facilities, and contextualizing cultural elements”.

## Data Availability

The raw data supporting the conclusions of this article will be made available by the authors, without undue reservation.
